# Urethral Microenvironment Adapted Sodium Alginate/Gelatin/Reduced Graphene Oxide Biomimetic Patch Improves Scarless Urethral Regeneration

**DOI:** 10.1002/advs.202302574

**Published:** 2023-11-16

**Authors:** Liyang Wang, Kai Wang, Ming Yang, Xi Yang, Danyang Li, Meng Liu, Changmei Niu, Weixin Zhao, Wenyao Li, Qiang Fu, Kaile Zhang

**Affiliations:** ^1^ The Department of Urology Shanghai Sixth People's Hospital Affiliated to Shanghai Jiao Tong University School of Medicine Shanghai Jiao Tong University Shanghai 200233 P. R. China; ^2^ School of Materials Science and Engineering Shanghai University of Engineering Science Shanghai 201620 P. R. China; ^3^ Clinical Research Center Shanghai Chest Hospital Shanghai Jiao Tong University Shanghai 200233 P. R. China; ^4^ Shanghai Eastern Institute of Urologic Reconstruction Shanghai 200000 P. R. China; ^5^ Novaprint Therapeutics Suzhou Co., Ltd Suzhou 215000 P. R. China; ^6^ Wake Forest Institute for Regenerative Medicine Winston‐Salem NC 27155 USA

**Keywords:** 3D printing, hydrogels, reduced graphene oxide, urethral stricture, urethral reconstruction

## Abstract

The nasty urine microenvironment (UME) is an inherent obstacle that hinders urethral repair due to fibrosis and swelling of the oftentimes adopted hydrogel‐based biomaterials. Here, using reduced graphene oxide (rGO) along with double‐freeze‐drying to strengthen a 3D‐printed patch is reported to realize scarless urethral repair. The sodium alginate/gelatin/reduced graphene oxide (SA/Gel/rGO) biomaterial features tunable stiffness, degradation profile, and anti‐fibrosis performance. Interestingly, the 3D‐printed alginate‐containing composite scaffold is able to respond to Ca^2+^ present in the urine, leading to enhanced structural stability and strength as well as inhibiting swelling. The investigations present that the swelling behaviors, mechanical properties, and anti‐fibrosis efficacy of the SA/Gel/rGO patch can be modulated by varying the concentration of rGO. In particular, rGO in optimal concentration shows excellent cell viability, migration, and proliferation. In‐depth mechanistic studies reveal that the activation of cell proliferation and angiogenesis‐related proteins, along with inhibition of fibrosis‐related gene expressions, play an important role in scarless repair by the 3D‐printed SA/Gel/rGO patch via promoting urothelium growth, accelerating angiogenesis, and minimizing fibrosis in vivo. The proposed strategy has the potential of resolving the dilemma of necessary biomaterial stiffness and unwanted fibrosis in urethral repair.

## Graphical abstract

A sodium alginate/gelatin/rGO biomimetic patch is created with printable, degradable, and adjustable mechanical properties. The inclusion of rGO with the appropriate concentration can promote cell migration, and proliferation, and inhibit urethral fibrosis and the formation of the urothelium. This strategy demonstrates great potential in addressing the dilemma of necessary biomaterial stiffness and unwanted fibrosis in urethral repair.

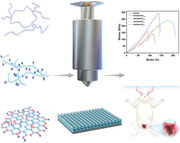

## 1 Introduction

Urethral stricture, which is a narrowing process of urethra lumen caused by fibrosis of the urethra mucosa and the surrounding spongy corpus spongiosum, is often inevitable after urethral injury.^[^ ^1^, ^2^, ^3^ ^]^ It has been reported that 229 to 627 out of 100 000 males suffer from urethral stricture, but the treatment is still limited in the field of urology.^[^ ^4^ ^]^ Obviously, urethral stricture has a substantial impact on the quality of life in patients with high health costs.^[^ ^4^ ^]^ Urethroplasty procedures have been considered to be the most effective gold‐standard method, compared to certain micro‐invasive surgeries (e.g., urethral dilation and internal urethrotomy).^[^ ^5^, ^6^ ^]^ A number of challenges indeed exist in the surgical treatment of urethral strictures, such as the use of substitute materials (autologous penile flap or oral mucosa).^[^ ^7^ ^]^ The autologous substitute materials for urethral reconstruction have limitations, which can cause damage at the harvesting site. Thus, suitable tissue engineering and alternative biomaterials have recently been intensively explored for more effective regenerative medicine in an attempt to overcome such problems of urethral reconstruction.^[^ ^5^, ^7^, ^8^, ^9^, ^10^ ^]^

It is difficult to mimic an actual urethral structure with the dense mucosal layer and the loose submucosal layer for scarless regeneration. 3D fabrication of biologically functional components is certainly a promising technology, as it adopts the extracellular matrix (ECM) molecules and provides a biomimetic microenvironment for cells.^[^ ^11^, ^12^ ^]^ With its emergence, the synthesis of scaffolds has attracted enormous attention owing to the programmable deposition of biocompatible materials.^[^ ^13^, ^14^ ^]^ Currently, hydrogel biomaterials are routinely used as (bio)inks in extrusion‐based 3D (bio)printing thanks to the printability and cell‐laden capacity.^[^ ^15^ ^]^ In our previous study, we have shown that the fibrin hydrogels laden with urothelial cells (UCs) and smooth muscle cells (SMCs) could maintain sufficient cell viability and proliferation.^[^ ^7^ ^]^ Nonetheless, the mechanical properties of fibrin were too weak to function as the urethral scaffold. Thus, a new class of hydrogel biomaterials for urethral reconstruction is strongly necessary.

SA and Gel, as the natural hydrogel biomaterials that are biocompatible and printable, have been widely used in engineered organs.^[^ ^16^, ^17^, ^18^ ^]^ There are some unique characteristics for SA and Gel. The polymer chains of SA can be cross‐linked with Ca^2+^ to form a stable structure for mechanical support.^[^ ^19^ ^]^ The microenvironment of urethra is filled with urine that has plenty of calcium ions.^[^ ^20^ ^]^ It is feasible to realize a second cross‐link of SA to reinforce the strength. Gel is a thermosensitive material that transforms from liquid phase to gel state via physical cross‐linking, and maintains the initial structural stability of the 3D‐printed scaffolds.^[^ ^21^ ^]^ Meanwhile, due to the arginine‐glycine‐aspartic acid (RGD) sequences intrinsic to the Gel chains, it is considered friendly to the enhanced cell attachment and growth.^[^ ^22^, ^23^ ^]^

For 3D printing, the formulation of the inks is the decisive factor for the successful scaffold fabrication.^[^ ^15^ ^]^ Zhang et al. have designed the low‐density SA (0.8%) and Gel (4.1%) hybrid bioinks for 3D bioprinting.^[^ ^14^ ^]^ The results showed that the cell viability for the concentration of 0.8% SA was 84 ± 0.7%. Yet, the mechanical properties, printability, and shape fidelity of the scaffolds were insufficient. The cell viability of 2.3% SA was only 68 ± 1.3%, but the fidelity and mechanical properties of the scaffolds were improved due to the increased viscosity of SA. The optimal concentrations of printability ranged from 0.6 to 2.5%, depending on the viscosity of SA. Several other studies have also optimized the formulation of SA and Gel to satisfy the printability of bioinks and improve the viability of cells.^[^ ^24^, ^25^, ^26^, ^27^ ^]^

On the other hand, to obtain the desired properties of bioinks, certain nanomaterials (e.g., inorganic and carbon nanomaterials) have been integrated with SA/Gel to prepare the nanocomposite bioinks.^[^ ^28^, ^29^ ^]^ Graphene‐based materials are considered to be the reinforcement nanomaterials: the representative derivatives are GO and rGO, with attributes of favorable antibacterial properties,^[^ ^30^, ^31^ ^]^ angiogenic potential,^[^ ^32^, ^33^ ^]^ mechanical strength,^[^ ^34^, ^35^ ^]^ as well as low cytotoxicity.^[^ ^36^ ^]^ Studies have suggested that regulating the surface oxygen content of rGO enhances the cell adhesion and proliferation due to the non‐covalent interactions to improve the surface adsorption of rGO.^[^ ^37^ ^]^ In addition, Mukherjee et al. reported that rGO exhibited considerable angiogenic activity in a dose‐dependent manner.^[^ ^32^ ^]^ Cell proliferation and wound healing were also promoted by the increased concentration of reactive oxygen species (ROS) due to the incorporation of rGO.^[^ ^38^, ^39^ ^]^

**Scheme 1:**
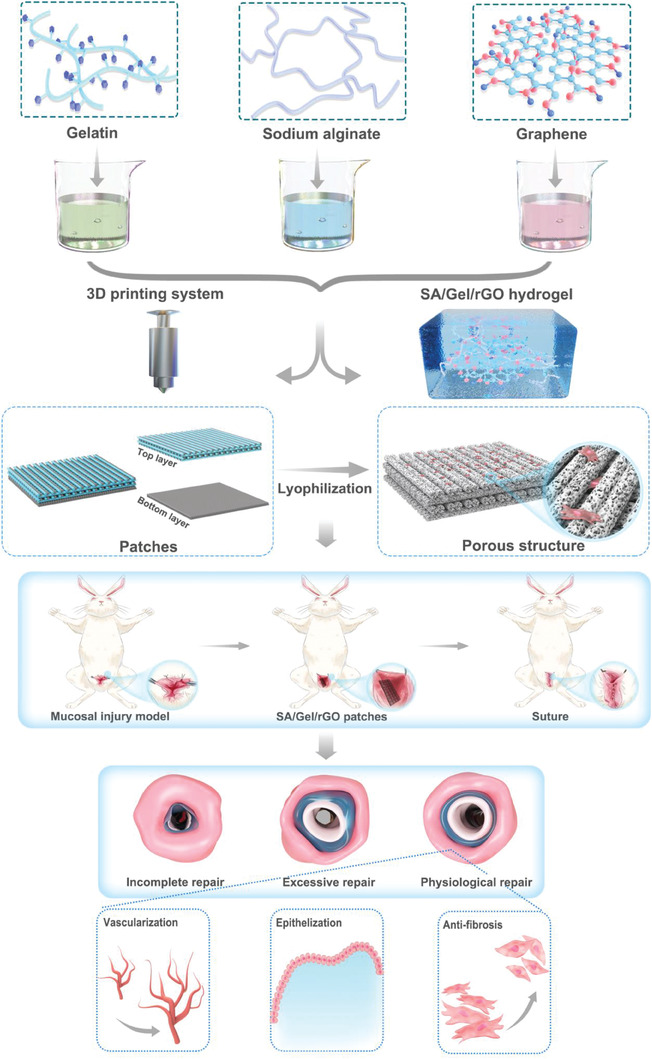
Schematics on the development of 3D‐printed SA/Gel/rGO patches for urethral repair. The SA/Gel/rGO patches were prepared by a 3D‐printing system and cross‐linked by Ca^2+^. The patches were then sutured in the damaged urethra of rabbits, wherein the rGO component facilitated regeneration of mucosa, promoted vascularization, and exhibited anti‐fibrotic activity.

To our knowledge, there are still few studies on fabricating the urethral substitutes by the 3D‐printing technology that would function in the harsh urethral microenvironment. The ideal treatment on urethral stricture should exhibit the following characteristics: i) the match of the patch with the structure of urethral decellularized matrix (Figure S1, Supporting Information); ii) angiogenesis and reconstruction of urethral functions; iii) inhibition of bioactive factors on urethral fibrosis and scar hyperplasia. In this study, we design a 3D‐printed SA/Gel/rGO patch, by a double‐freeze‐drying process and also cross‐linked by Ca^2+^ with enhanced stability, to characterize its anti‐fibrotic, angiogenic, and epitheliogenic properties in urine (**Scheme** **1**). Systematic experiments in vitro and in vivo were conducted to understand the unique features, which would demonstrate the therapeutic efficacy of the patch for urethral reconstruction.

## 2 Results and Discussion

### 2.1 rGO Characterizations and Ink Preparation

**Figure 1:**
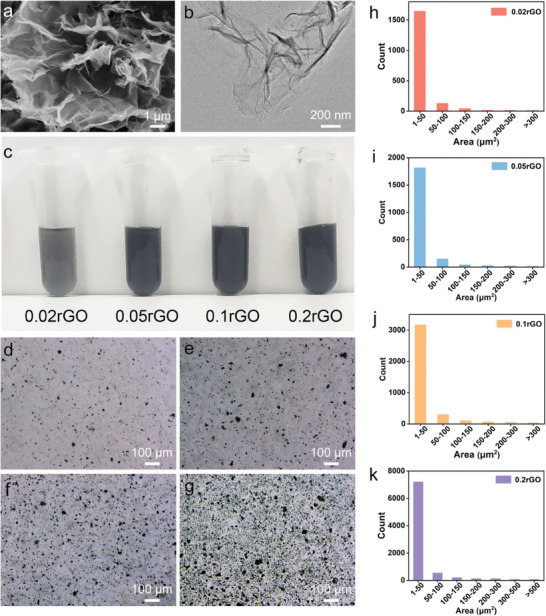
Morphologies of rGO and optical images of rGO/hydrogel‐precursor dispersions. a) SEM and b) TEM images of rGO. c) Photographs of different concentrations of rGO/hydrogel‐precursor suspensions at rGO concentrations of 0.02, 0.05, 0.1, and 0.2 mg mL^−1^. d–g) Corresponding optical microscopy images. h–k) Area counts of the rGO distributed in the corresponding suspensions; *p <0.05, ****p <0.0001, n.s. means not significant.

Commercially available monolayer rGO powder was used. The rGO was dispersed in water according to the information provided by the manufacturer. The monolayer ratio of rGO powder was up to 80%, the diameters were 0.5–5 µm, and the thicknesses were 0.8–1.2 nm. The morphology of rGO was confirmed under scanning electron microscope (SEM) and transmission electron microscope (TEM) (**Figure** **1**a,b). The stock rGO dispersion (1 mg mL^−1^) was diluted to concentrations in a series of 0.02, 0.05, 0.1, and 0.2 mg mL^−1^, and all suspensions remained stable and homogeneous for several weeks upon evaluation. With the increasing concentration of rGO, the solution color changed from gray to dark (Figure 1c). The solutions were also examined under optical microscopy to show the good dispersion with no obvious aggregation for rGO (Figure 1d–g). The area distributions of the rGO were mostly at the 1–50 µm^2^ range (Figure 1h–k). The mean area rate (%) is from 4% to 28% (Figure S2, Supporting Information). The results demonstrate that the rGO distributes evenly in the water solution, and most of the rGO particles do not adhere to each other.

### 2.2 Characterizations of SA/Gel/rGO Hydrogels

**Figure 2:**
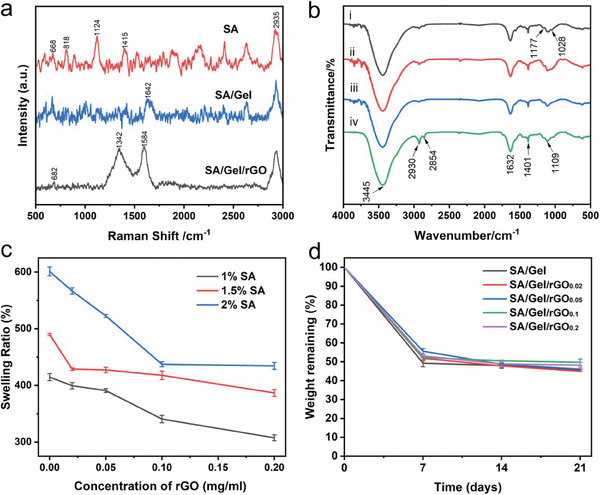
Physicochemical characterizations of the SA/Gel/rGO hydrogels. a) Raman spectra of SA, SA/Gel, and SA/Gel/rGO hydrogels. b) FTIR spectra of i) SA/Gel hydrogel, ii) SA/Gel/rGO_0.02_ hydrogel, iii) SA/Gel/rGO_0.05_ hydrogel, and iv) SA/Gel/rGO_0.2_ hydrogel. c) Swelling ratios of the different concentrations of SA and SA/Gel/rGO composite inks at different concentrations of rGO. d) Degradation profiles of the hydrogels.

Raman spectroscopic analysis of SA/Gel/rGO shows two characteristic bands of G‐ and D‐bands (**Figure** **2**a). The D‐band was at ≈1342 cm^−1^ and the G‐band was at ≈1584 cm^−1^. The D‐band reflects the disorder between graphite lamellae due to the interference of *sp^2^* hybridization of carbon. The G‐band reflects the symmetry and degree of crystallization due to the *sp^2^* hybrid in‐plane stretching vibration of carbon crystals. I_D_/I_G_ is the intensity ratio between the D‐ and G‐bands, suggesting the degree of defects in the graphite lamellae^[^ ^40^, ^41^ ^]^; the higher the intensity ratio is, the more defects the C atomic crystal has. Chakraborty reported that the ID/IG ratios of GO and rGO were 0.9 and 1.1, respectively.^[^ ^42^ ^]^ To verify the incorporation of rGO into the SA/Gel hydrogels, the composition of the SA/Gel/rGO composite was confirmed through Fourier‐transform infrared (FTIR) spectroscopy (Figure 2b). The SA hydroxyl bond (O─H) stretching was at ≈3445 cm^−1^, the CH_2_ group stretching was at ≈2935 cm^−1^, and the carbonyl bond (C═O) stretching of amide I was at 1632 cm^−1^. The characteristic bands of SA at ≈1632 and 1401 cm^−1^ are associated with the carboxyl (─COOH) bond and asymmetric and symmetric stretching peaks of carboxylate salt groups, respectively. The peak at 1542 cm^−1^ is the ─NH group stretching of amide II and the peak at 1238 cm^−1^ is the ─NH group stretching of amide III of Gel. The peak at 1177 cm^−1^ is the C─O─C asymmetric stretching. The peak at 1028 cm^−1^ is the C─O─H stretching of SA. The characteristic peaks of rGO at ≈2930 and 2854 cm^−1^ are associated with CH_2_ and CH_3_ groups, respectively. Interestingly, the strong absorption peaks at ≈2930 and 2854 cm^−1^ appeared with the increased concentration of rGO. As such, the FTIR spectra clearly indicated the presence of the rGO in the hybrid hydrogels.

The swelling behavior of a hydrogel construct plays a crucial role in its surface properties and mechanical integrity for biomaterial applications.^[^ ^43^, ^44^ ^]^ The swelling behaviors of the various hydrogel formulations suggested that the swelling ratio increased when the concentration of SA was elevated (1%, 1.5%, and 2%) and the concentration of rGO was reduced (Figure 2c). The higher content of SA enhanced the hydrophilicity of the hydrogel network to improve the equilibrium swelling ratio of SA. For the positive effect of rGO concentration, it was likely due to the ability of rGO to interact with the hydrogen bonds of SA/Gel to act like a multi‐functional cross‐linking agent, therefore increasing the density of the cross‐linked network of the SA/Gel hydrogels at a higher concentration.

Overtime, the hydrogels would be subjected to hydrolysis and other forms of degradation after being immersed in the liquid environment. The degree of degradation was evaluated by the mass remaining (%) of the hydrogels over 21 days. The various degrees of degradation showed a significant weight reduction in the first 7 days (Figure 2d), which might be due to the hydrolysis of the hydrogels. The remaining mass percentages (%) of the hydrogels are ≈42.62 ± 1.98% to 55.58 ± 1.44% in the different groups. In the next few days, there was a slight decrease in the weights of the hydrogels, which might correspond to the reduced degradation of the cross‐linked structure. The total weights of hydrogels had no significant differences between the SA/Gel hydrogels with and without rGO, implying that the incorporation of rGO did not noticeably affect the degradation of the hydrogel formulations.

### 2.3 Morphological Evaluations on the SA/Gel/rGO Hydrogels

**Figure 3:**
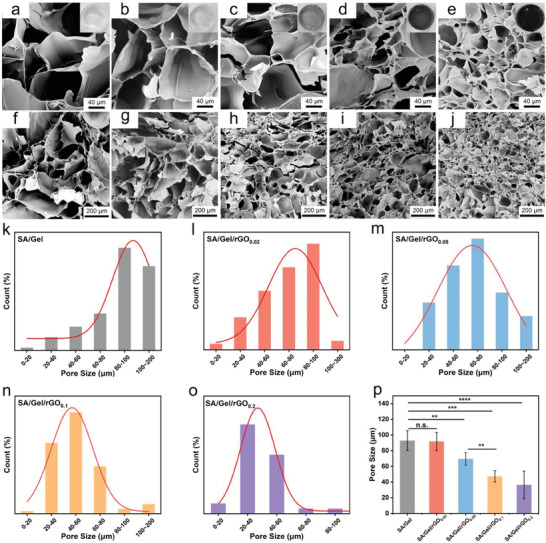
SEM images and gross appearances of SA/Gel/rGO hydrogels with various concentrations of rGO: 0, 0.02, 0.05, 0.1, and 0.2 mg mL^−1^. a–e) SEM images at 1000× magnification and optical images of the hydrogels; f–j) SEM images at 300× magnification. k–o) Distributions of pore sizes of the different hydrogels. p) Average pore sizes of the different hydrogels; **p <0.01; ***p <0.001; ****p <0.0001; n.s. means not significant.

The colors of the SA/Gel/rGO hydrogels were observed to be darker with the increased concentration of rGO. The SEM images reveal the porous structures of the SA/Gel/rGO hydrogels with different concentrations of rGO (0, 0.02, 0.05, 0.1, and 0.2 mg mL^−1^) (**Figure** **3**a–j). As the rGO loading increases, the pore size decreases and the pore density increases, further indicating that a high rGO concentration led to a high cross‐linking density, resulting in smaller pore sizes in composite hydrogels (Figure 3b–j). The pore size of hydrogels is highly correlated with cell adhesion, growth and proliferation, and nutrient exchange in the matrix. Most importantly, the hydrogels with a porous structure could facilitate efficient biomolecule transport within the environment.^[^ ^42^ ^]^ However, it should be noted that the pore sizes obtained by SEM observations may not indicate the actual pore sizes of the hydrogels under the hydrated states.

From the distributions of the pore sizes for the SA/Gel/rGO hydrogels (Figure 3k–o), there were network structures with the pore sizes of <100 µm for all the hydrogels. The distributions of the pore sizes of the SA/Gel hydrogel were mainly in the range of 80–200 µm; the pore sizes of the SA/Gel/rGO hydrogels decreased with the increasing concentration of rGO (Figure 3p). The pore sizes of the SA/Gel and SA/Gel/rGO_0.02_ hydrogels were 92.82 ± 12.60 and 91.59 ± 11.6 µm, respectively . There was significant difference between SA/Gel/rGO_0.05_ (69.39 ± 7.97 µm) and SA/Gel/rGO_0.1_ (47.20 ± 7.26 µm), compared with the SA/Gel hydrogels. The pore size of SA/Gel/rGO_0.2_ was the smallest among all the hydrogels (36.29 ± 17.77 µm, ). These results showed that the incorporation of rGO indeed influenced the pore size of the hydrogels in a dose‐dependent pattern.

### 2.4 Morphological and Mechanical Evaluations on SA/Gel/rGO Patches

**Figure 4:**
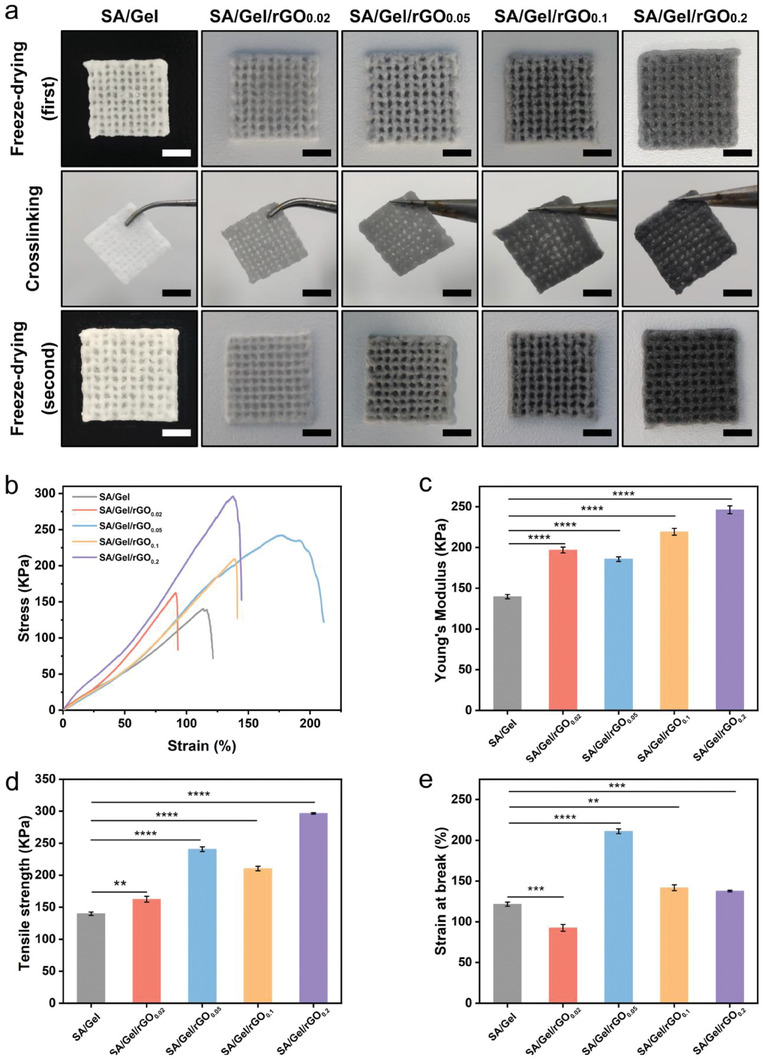
a) Morphology of the 3D‐printed SA/Gel/rGO patches containing different concentrations of rGO, and the double‐freeze‐drying process. b) Tensile stress‐strain curves; c) Young's moduli; d) tensile strengths at break; and e) strains at the break for the different 3D‐printed patches; **p <0.01; ***p <0.001; and ****p <0.0001.

We 3D‐printed various SA/Gel scaffolds with different concentrations of rGO from 0 to 0.2 mg mL^−1^ (**Figure** **4**a). We used two freeze‐drying and one cross‐linking process to obtain the patches post‐printing. The gross morphologies were not significantly different except that the color changed from white to dark at the rGO concentration was increased. The 3D printer was an Organ Printing United system (Figure S3a, Supporting Information). The mechanical properties of the SA/Gel/rGO patches are a valuable element for urethral repair and tissue regeneration. The tensile mechanical characteristics of the SA/Gel/rGO patches with different concentrations of rGO were evaluated by the stretching process of the patches (Figure S3b, Supporting Information), the length of the patches that stretched between the upper and lower grips of the tensile machine could be adjusted. Then, each sample was stretched at 10 mm per minute until reaching its breaking points. All the samples exhibited the linear elastic behavior (Figure 4b). Compared with the patches containing rGO, the SA/Gel patch without rGO showed a lower modulus, 139.68 ± 0.144 KPa (Figure 4c). The tensile strengths of the SA/Gel patches at concentrations of 0, 0.02, 0.05, and 0.2 mg mL^−1^ of rGO were in the range of 139.87 ± 1.02–296.65 ± 1.11 KPa (Figure 4d). The tensile strength of the SA/Gel/rGO_0.2_ patch was higher (296.65 ±1.11 KPa) when the concentration of rGO was increased. The elevation of strain at break in the SA/Gel/rGO_0.05_ was 211.10 ± 1.34% (Figure 4e). Besides, the patches with appropriate elongation are flexible and elastic. Interestingly, the strain at break of SA/Gel/rGO_0.2_ patch was 137.77 ± 0.86% . These observations might be attributed to the SA/Gel patch to become fragile under the higher concentration of rGO. The SA/Gel/rGO patch was saturable and exhibits excellent mechanical properties to fit the requirement of urethral reconstruction. Interestingly, the rGO hybrid patches appeared to possess higher tensile strengths and Young's modulus. The rGO_0.05_ was an appropriate concentration to achieve the best mechanical performance among the groups examined.

### 2.5 Characteristics of Cell Behaviors

**Figure 5:**
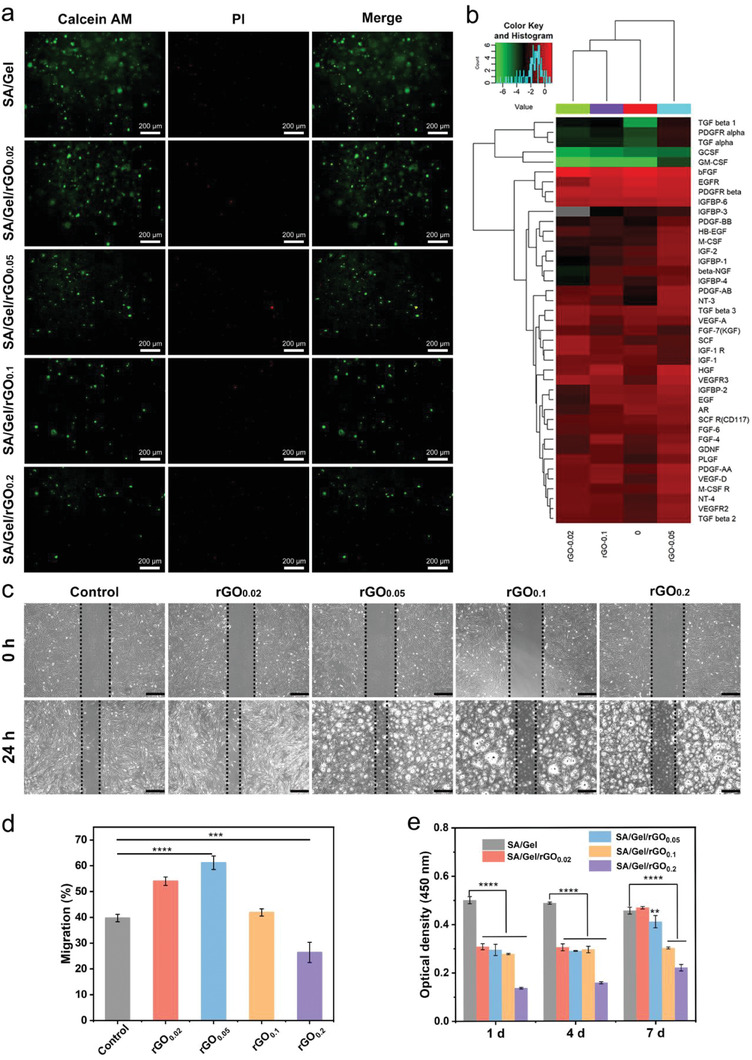
In vitro cytocompatibility of rGO and rGO composite hydrogel. a) Cell viability of fibroblasts seeded on different concentrations of SA/Gel/rGO hydrogels at day 1. Living cells are depicted in green and dead cells in red. b) Heat map of DEPs. Red and green colors indicate high and low expressions, respectively. c) wound healing was observed in fibroblast cells treated with the different concentrations of rGO (0, 0.02, 0.05, 0.1, and 0.2 mg mL^−1^). fibroblast cells treated without rGO were used as a positive control experiment. The scale bar at the right lower corner is 500 µm. d) The percentage of wound contraction was measured using Image J analysis software. Statistical significance was calculated by using a t‐test. e) Cell proliferation of fibroblasts cultured on hydrogels for 1, 4, and 7 days; n = 6, **p <0.01, ***p <0.001, ****p <0.0001.

SA/Gel/rGO patches served as the templates for cell adhesion, and proliferation, thus superior cytocompatibility is required. The characteristics of cell survival and proliferation were evaluated through seeding fibroblasts on SA/Gel/rGO hydrogels containing different concentrations of rGO. The live and dead cells were stained with calcein AM and propidium iodides (PI) after 1 day, respectively. The fluorescence of living cells in SA/Gel/rGO hydrogels of 0.1 and 0.2 mg mL^−1^ of rGO was less (**Figure** **5**a). The increased number of dead cells might have been affected in part by the relatively high concentrations of rGO present. The cell morphologies were further observed by SEM, where fibroblasts were generally located within the pores of the 3D‐printed SA/Gel/rGO patches (Figure S4, Supporting Information).

From the expression patterns of differentially expressed proteins (DEPs) by fibroblasts (cell density: 1 × 10^7^) with the different concentrations of rGO (Figure 5b), the cells treated with rGO of 0.02, 0.05, and 0.1 mg mL^−1^ were compared with the cells with no treatment. The culture time was 72 h. Some biological processes and pathways, which are correlated with tissue regeneration or tissue repair, were observed to be significantly enriched in these DEPs (Figures S5a,S6a, and S7a, Supporting Information). For example, it is reported that the transmembrane receptor protein tyrosine kinase signaling pathway has a role in promoting sprouting of the angiogenic endothelium.^[^ ^45^ ^]^ The Ras/MAPK signaling cascade plays an important role in zebrafish heart regeneration.^[^ ^46^ ^]^ The cell proliferation process is required for cardiomyocytes regeneration.^[^ ^47^ ^]^ In addition, the cellular components related to platelet alpha granule, secretory veside, vesicle lumen, and endomembrane system were enriched in DEPs treated with rGO_0.02_ (Figure S5b, Supporting Information). Similar situations were observed when treated with rGO_0.05_ and treated with rGO_0.1_ (Figures S6b and S7b, Supporting Information). In addition, in molecular functions, the growth factor binding, growth factor activity, receptor‐ligand activity, and signaling receptor activator were enriched (Figures S5c,S6c, and S7c, Supporting Information). Similarly, the enriched Ras and MAPK signaling pathways were also observed via the Kyoto Encyclopedia of Genes and Genomes (KEGG) pathway analysis (Figures S5d,S6d, and S7d, Supporting Information). The genes involved in these biological processes and pathways might contribute to angiogenesis mediated by rGO. The bubble graph was also shown for gene ontology enrichment treated with rGO of 0.02, 0.05, and 0.1 mg mL^−1^ for the biological process, molecular process, and cellular components (Figure S8a–i, Supporting Information).

Scratch assay is a standard for cell migration study in vitro. The migration of fibroblasts for the wound healing process is of great importance; after the injury, fibroblasts were used to proliferate and migrate from neighboring tissues into the wound area.^[^ ^48^ ^]^ To evaluate the potential of rGO in enhancing the migration of fibroblasts, a time‐dependent experiment was carried out (0–24 h). The results of the scratch assay showed that the migration ability of fibroblasts was greatly improved within 24 h after the treatment with rGO concentrations of 0.02 and 0.05 mg mL^−1^ (Figure 5c). With the increasing concentration of rGO, the proliferation of fibroblasts and wound healing increased first and then decreased, with a maximum migration rate of 61.18 ± 2.61% for rGO at 0.05 mg mL^−1^ (Figure 5d). Of note, when the concentration of rGO was set at 0.2 mg mL^−1^, the migration and proliferation of fibroblasts were significantly inhibited (26.39 ± 3.93%).

Cell counting kit‐8 (CCK‐8) assay was subsequently used to assess the cytotoxic effect of SA/Gel/rGO patches on the fibroblasts (Figure 5e). After 1 and 4 days of cell culture, the metabolic activities of cells on the SA/Gel/rGO patches exhibited inhibitory effects. The higher concentration of rGO revealed inhospitable environments to cell metabolic activities during this time point. However, after 7 days of cell culture, the cell metabolic activities on SA/Gel/rGO_0.02_ and SA/Gel/rGO_0.05_ became higher than the remaining patches close to that for the control patches without rGO. Consequently, it might be undesirably toxic to cells at the concentration of rGO above 0.05 mg mL^−1^, in alignment with the cell viability staining results. Some reports have suggested that GO concentration of 50 µg mL^−1^ on fibroblasts led to significant cytotoxicity, while rGO at a concentration higher than 0.1 µg mL^−1^ would be toxic to endothelial cells.^[^ ^32^, ^49^ ^]^ Our results were overall consistent with the previous studies.

### 2.6 Urethrogram and Gross Morphology

**Figure 6:**
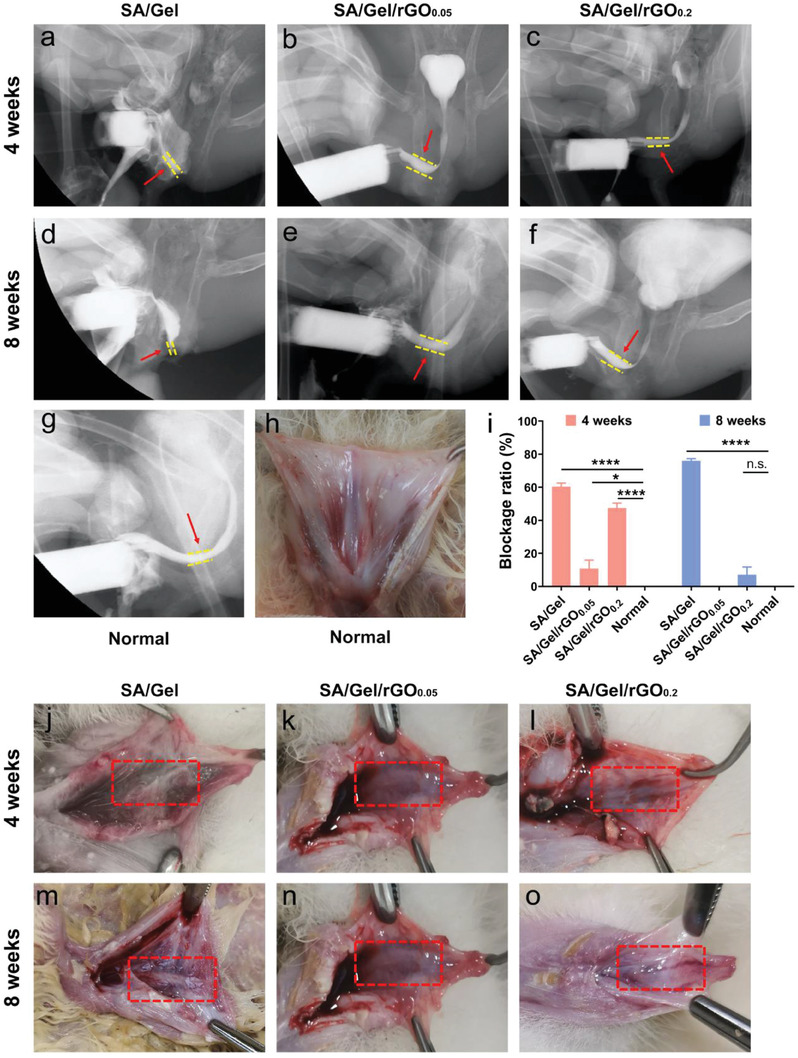
Urethrography examinations and appearances of urethral repair from the biomaterial with SA/Gel, SA/Gel/rGO_0.05_, and SA/Gel/rGO_0.2_. The results of urethras from a,d,g,j) SA/Gel, b,e,h,k) SA/Gel/rGO_0.05_, and c,f,i,l) SA/Gel/rGO_0.2_ at 4‐ and 8‐weeks post‐surgery. The red arrows indicate the lumen of the urethra. The red dotted boxes indicate the urethral epithelial layer. *p <0.05, ****p <0.0001, n.s. means not significant.

The safety and functions of the SA/Gel/rGO patches were further proven in clinically relevant rabbit urethral injury models. According to the results of the in vitro mechanical test and biocompatibility of the scaffold, the patches without rGO were selected as the control group, and the rGO concentration of 0.05 and 0.2 mg mL^−1^ was selected as the experimental group. The patches were sutured to the dorsal urethral defect, and the injury model of the urethra and the surgical process of repair are shown in Figure S9 (Supporting Information). The recovery of the repaired urethras was detected at 4 and 8 weeks after the operation. The 4‐ and 8‐week urethrogram of the SA/Gel group displayed narrow lumens because of scar formation (**Figure** **6**a,d). The SA/Gel/rGO_0.05_ (Figure 6b,e) groups showed wide lumens of urethras similar to the normal urethra (Figure 6g). In the 4‐week urethrogram, the SA/Gel/rGO_0.2_ groups had a narrower urethral lumen, the widths developed larger at 8 weeks (Figure 6c,f). The reason might be the inflammation‐related swelling of urethral tissue caused by rGO with high concentration. The blockage rate determines whether and how much urine can flow out. The blockage ratio of the urethras treated with the SA/Gel/rGO_0.05_ patches could approach that of normal lumens (Figure 6i). After the incision of the ventral urethral wall, the dorsal wall could be exposed. Gross morphology revealed that urethral tissue in the SA/Gel/rGO_0.05_ and SA/Gel/rGO_0.2_ groups was significantly better than that in the SA/Gel group. The SA/Gel group showed the growth of severe scar‐like tissue in the urethra (Figure 6j,m). At 8 weeks, both of SA/Gel/rGO_0.05_ (Figure 6n) and SA/Gel/rGO_0.2_ (Figure 6o) groups showed smooth epithelial layer similar to the normal urethra of rabbits (Figure 6h). However, SA/Gel/rGO_0.2_ group at 4 weeks (Figure 6i) revealed red and swollen mucosa compared with that in the SA/Gel/rGO_0.2_ group (Figure 6k), which is consistent with the lumen width in the urethrogram. Therefore, the treatment of urethral injury could be improved by choosing a low dose of rGO for future application.

### 2.7 Histological Evaluations

**Figure 7:**
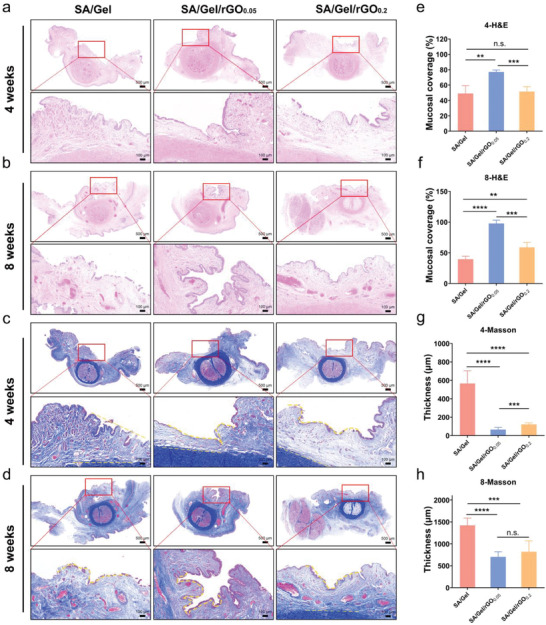
Histological examination of the regenerated tissues at the damaged urethra sites after staining the sections with H&E and Masson's Trichrome. Representative images of urethral cross‐sections of the reconstructed urethra using SA/Gel, SA/Gel/rGO_0.05_, and SA/Gel/rGO_0.2_ of a,c) at 4 weeks and b,d) at 8 weeks. The mucosal coverage of urethra at 4 weeks (a) and at 8 weeks (b); The collagen thickness of urethra at 4 weeks (a) and at 8 weeks (b). The green dotted lines indicate the border line of collagen thickness. Data are means ± standard error, *n* = 3; **p <0.01, ***p <0.001, ****p <0.0001, n.s. means not significant.

The histology analyses of urethral reconstruction at 4‐ and 8‐weeks post‐surgery were performed by hematoxylin and eosin (H&E) and Masson's trichrome staining. The SA/Gel patches with/without rGO showed various outcomes. Additionally, there was no significant infection during the experimental period, suggesting good biocompatibility of the patches. According to the histology, the urethras treated with the SA/Gel patches were associated with thinner/uncomplete urothelium layers, fewer blood vessels, and thicker submucosal tissue (**Figure** **7**a–d). Due to the lack of regenerated urothelium layers, and over‐deposited ECM, the urethra developed to urethral stricture. In contrast, the reconstructed urethra treated with the SA/Gel/rGO patches had the nearly normal urothelium layers, submucosal tissue, and blood vessels. The continuous and complete urothelium layers were formed on the lumen surfaces for the SA/Gel/rGO_0.05_ group after 4‐ and 8‐weeks post‐surgery. However, the SA/Gel/rGO_0.2_ patches could lead to a short period of swelling and inflammation in 4 weeks. These results suggested that rGO at a lower concentration (0.05 mg mL^−1^) could perform best function in the regeneration of urothelium layers and blood vessels, but not at a higher concentration (0.2 mg mL^−1^). The mucosal coverage ratios by the SA/Gel/rGO patches were measured (Figure 7e,f). The urethras treated with the SA/Gel/rGO_0.05_ patches had more significant coverages, compared with the other groups. The urethral collagen thicknesses were also measured after the wound healing (Figure 7g,h). The SA/Gel/rGO_0.05_ patch could induce a less collagen deposition under the mucosa, therefore developing a scar‐less wound healing. The histology showed that an optimal concentration of rGO would promote the regeneration of epithelial cells and decrease submucosal ECM deposition to inhibit urethral fibrosis.

### 2.8 Immunofluorescence

**Figure 8:**
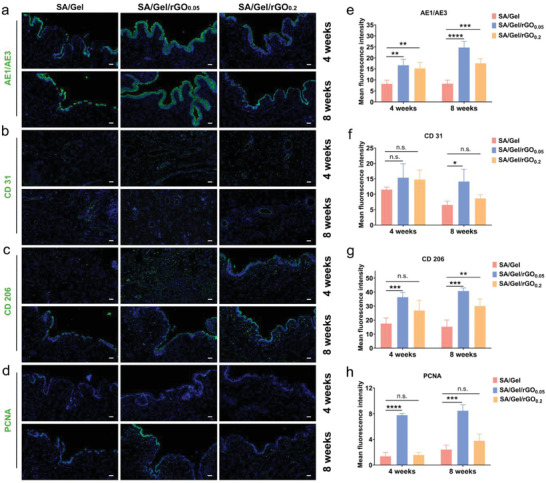
Immunofluorescence staining of specific biomarkers in the SA/Gel, SA/Gel/rGO_0.05_, and SA/Gel/rGO_0.2_ repair after 4‐ and 8‐weeks post‐surgery. Representative immunofluorescence images of a) AE1/AE3, b) CD31, c) CD206, d) PCNA. Scale bar: 50 µm. Blue: Nuclei. e) Quantification of expression levels of epithelial cytokeratin AE1/AE3, f) the blood vessels based on the CD 31, g) the inflammation based on CD206 (M2 macrophages), and h) cell proliferation based on PCNA. Data are mean ± standard error, *n* = 3; *p <0.05, **p <0.01, ***p <0.001; ****p <0.0001, n.s. means not significant.

The distribution of cytokeratin was investigated based on the expression of epithelial cytokeratin AE1/AE3 (an important membrane surface protein marker of ECs) via immunofluorescence staining of the urethra. The expression level for AE1/AE3 on the SA/Gel/rGO_0.05_ at 4‐and 8‐weeks post‐surgery (4W: 16.70 ± 2.66 and 8W: 24.72 ± 2.81) is significantly higher than that in the other groups. Although the continuous urothelium layer is formed, the urothelium layer of the SA/Gel/rGO_0.2_ group is thinner than that of the SA/Gel/rGO_0.05_ group. The expression of cytokeratin in the SA/Gel group is low (4W: 8.19 ± 1.72 and 8W: 8.29 ± 1.65), there is no complete regeneration of a discontinuous urothelium layer (**Figure** **8**a,e). It suggests that the introduction of rGO has the potential to promote the regeneration of urothelium layers. Studies have shown that the significant pro‐angiogenic properties of rGO depend on its concentration.^[^ ^32^, ^50^ ^]^ In the results of fluorescence staining at 4‐weeks post‐surgery, the SA/Gel/rGO_0.05_ group showed a number of CD 31‐positive cells (the blood vessels labeled by CD 31), which were more than them in the SA/Gel and SA/Gel/rGO_0.2_ group. At the beginning process of urethral regeneration with inflammation, the vessels in tissue would be much more obvious that is consistent with the results.^[^ ^51^ ^]^ For SA/Gel/rGO_0.05_ and SA/Gel/rGO_0.2_ groups, the number of CD 31‐positive cells at 8 weeks (SA/Gel/rGO_0.05_: 14.10 ± 3.99 and SA/Gel/rGO_0.2_: 8.63 ± 1.23) are lower than them at 4 weeks (SA/Gel/rGO_0.05_: 15.35 ± 4.55 and SA/Gel/rGO_0.2_: 14.77 ± 3.07) (Figure 8b,f).

Different degrees of inflammation response at the injured sites would appear because of the transplantation of external scaffolds. CD 206 is used to label M2 macrophages.^[^ ^52^ ^]^ The results showed that the number of CD206‐positive cells in the SA/Gel group was the lowest. The number of CD206 positive cells in the SA/Gel/rGO_0.05_ group in 4‐and 8‐weeks post‐surgery (4W: 36.36 ± 3.54 and 8W: 40.74 ± 2.23) were significantly higher than them in the other groups, indicating that the lower level of local inflammatory response was caused by the rGO treated group (Figure 8c,g). This indicated that rGO appeared to promote the transition of M2 macrophages and inhibit the inflammatory response during the urethral repair. Meanwhile, the low inflammatory response might promote the proliferation and activation of fibroblasts. The positive expression of proliferating cell nuclear antigen (PCNA) was obviously increased in the SA/Gel/rGO_0.05_ group at 4‐and 8‐weeks post‐surgery (4W: 7.77 ± 0.25 and 8W: 8.46 ± 0.94) (Figure 8d,h).

### 2.9 Flow Cytometric Analysis of RAW264.7 Surface Markers

**Figure 9:**
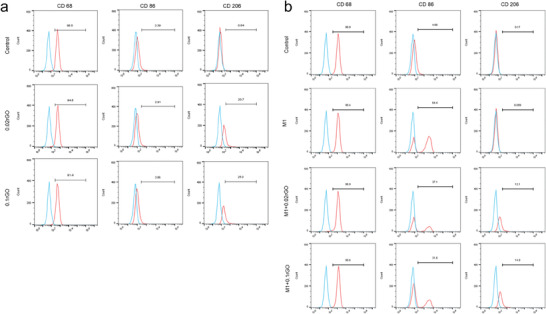
Flow cytometric analysis of RAW264.7 surface markers (CD68, CD86, and CD206). a) group 1 (Control; 0.02rGO; 0.1rGO). b) group 2 (Control; M1; M1 + 0.02rGO; M1 + 0.1rGO), where, M1; M1 + 0.02rGO; M1+ 0.1rGO, first treated with LPS (100 ng mL^−1^) + INF‐r (50 ng/mL) for 24 h to induce M1 type.

To evaluate the macrophages polarization status, the M1 surface markers (CD68, CD86) and M2 surface markers (CD206) in RAW 264.7 macrophages were further analyzed by flow cytometric (**Figure** **9**a). The results revealed that more than 90% of the cells strongly expressed surface antigens such as CD68. The inflammatory cell density was evaluated by CD86 staining. The CD86 inflammatory cell density in the 0.02rGO and 0.1rGO groups was no significant difference in the control group. However, as the amount of rGO increases, it leads to the increase of CD206‐positive macrophages. The expression of CD86 in M1 type was significantly increased by LPS + INF‐r, and the expression of CD206‐positive macrophages was significantly higher than that in the control group after the introduction of rGO (Figure 9b). The results of flow cytometry analysis showed that the addition of rGO promoted the polarization of macrophages to the M2 phenotype in vivo.

### 2.10 qRT‐PCR Analysis of mRNA Levels of the Markers in Tissue and RAW 264.7 Cells

**Figure 10:**
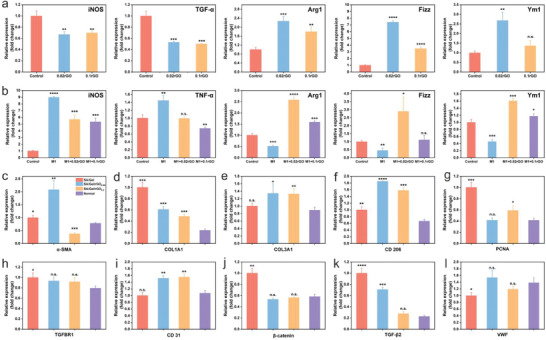
qRT‐PCR analysis of mRNA levels of the indicated macrophage markers in rGO co‐cultured with the RAW 264.7 cells and/or factors. The gene expression levels of a) iNOS, TGF‐α, Arg‐1, Fizz, Ym1, and b) iNOS, TGF‐α, Arg‐1, Fizz, Ym1. qRT‐PCR analysis of mRNA levels of the markers and factors in tissue. The gene expression levels of c) α‐SMA, d) COL1A1, e) COL3A1, f) CD206, g) PCNA, h) TGFBR1, i) CD31, j) β‐catenin, k) TGF‐β2, l) VWF. *p <0.05, **p <0.01, ***p <0.001, ****p <0.0001, n.s. means not significant.

The gene expression of Arg1, Fizz, and Ym1 in the 0.02rGO group and the 0.1rGO group was significantly higher than that in the control group, and the gene expression of iNOS and TGF‐β in the 0.02 rGO group and the 0.1rGO group was significantly lower than that in the control group (**Figure** **10**a). At the mRNA level, rGO treatment was associated with significant increases in Arg1, Fizz, and Ym1 expression compared to the control and M1 groups, and the gene expression of iNOS and TGF‐β in the 0.02 rGO group and the 0.1rGO group was significantly lower than that in the M1 group (Figure 10b). These results suggest that rGO may also have an inducing effect on the macrophage anti‐inflammatory phenotype in vivo. The expression of SA/Gel/rGO_0.05_ α‐SMA, COL1A1, COL3A1, CD206, CD31, TGF‐β2 was significantly higher than that in the control group. The expression of TGFBR1, β‐catenin, and VWF was no significant difference in the control group (Figure 10c–i). Overall, the low‐dose rGO effectively promoted epithelization and neovascularization while reducing inflammation. These results suggested that rGO can promote urethral healing and regeneration.

## 3 Conclusion

In this study, the SA/Gel/rGO patches were synthesized by 3D printing with printable, degradation, and tunable mechanical features for urethral reconstruction. Our results were interesting in that the physical and biological properties of SA/Gel/rGO patches were controllable by adjusting the concentration of rGO. rGO with proper intermediate concentrations turned out to promote cell migration, proliferation, and urothelium layer formation. In addition, the fibrosis of the urethra was inhibited by introducing low‐dose rGO. The urethral reconstruction in rabbits model using the optimized SA/Gel/rGO_0.05_ patches led to the scar‐less urethral regeneration. Totally, our study provided a systematic evaluation of the application potential of rGO‐based scaffold on the urological and other organ defects’ treatment.

## 4 Experimental Section

### Inks preparation and rGO characterization

The rGO was dispersed (1 mg mL^−1^) in water, and the solution was prepared according to the instructions. Briefly, the graphene powder (rGO, 20 mg, XFNANO, Nanjing, China) was dispersed in 20 mL double‐distilled water. And the aqueous suspension was obtained by ultrasonication at 40 KHz for 2 h. Polyvinyl pyrrolidone (PVP, MW = 58 000, 100 mg, Aladdin, Shanghai, China) was added to rGO aqueous suspension for sonication at ambient temperature for 2 h. After that, the rGO suspension was diluted to 0.02, 0.05, 0.1, and 0.2 mg mL^−1^, respectively.

SA powder (2% weight/volume, from brown algae, Sigma–Aldrich, USA) and Gel powder (2% w/v, from porcine skin, Tape A, gel strength ≈300 g Bloom, Sigma–Aldrich, USA) were added to sterile water to prepare the 0 rGO group as a control. Five GA/Gel/rGO hybrid ink solutions containing different rGO concentrations, 0, 0.02, 0.05, 0.1, and 0.2 mg mL^−1^ rGO, were named as SA/Gel, SA/Gel/rGO_0.02_, SA/Gel/rGO_0.05_, SA/Gel/rGO_0.1_, and SA/Gel/rGO_0.2_, respectively. To keep the same SA and Gel concentrations, different volumes of sterile water and rGO dispersion were prepared. The amounts of chemicals used for preparing the different SA/Gel/rGO hybrid ink solution are listed (Table S1, Supporting Information). As an example, to prepare the SA/Gel/rGO_0.05_ ink solution, 1.0 mL rGO solution (1 mg mL^−1^) was mixed with 19 mL sterile water to a homogeneous solution. Then, 400 mg SA and 400 mg Gel were dissolved in the above solution at 37 °C for 24 h. The mixture was kept standing to homogenize, sufficiently dissolve the polymer, and eliminate air bubbles. The rGO morphology was studied by using TEM (FEI, Hillsboro, USA) and SEM (Hitachi TM‐100, Tokyo, Japan). The spatial distribution of rGO was observed under an inverted phase contrast microscope (Olympus, Japan).

### Characterizations of SA/Gel/rGO hydrogel

1. *Raman Shift*: The rGO incorporation into the hydrogel was analyzed qualitatively via Raman spectroscopy (DXR 2Xi, ThermoFisher, United States).
2. *Fourier‐Transform Infrared (FTIR)*: The functional groups of hydrogels were analyzed by FTIR (iS50, ThermoFisher, United States). The hydrogel was freeze‐dried in a vacuum freeze‐dryer (SCIENTZ‐10N, Ningbo, China) to obtain the powder. The hydrogel powder was finely grounded with KBr and compressed into slices for FTIR measurements, over the range of 500–4000 cm^−1^.
3. *Swelling Ratio*: The swelling property was assessed after immersion in double‐distilled water for 5 days by the general gravimetric method, which was referred to by Liu.^[^ ^53^ ^]^ Three different concentrations of SA (1%, 1.5%, and 2%) and rGO hybrid hydrogel were investigated (*n* = 4 per group). The freeze‐dried hydrogels samples were weighed (*W*_d_) and placed in a 24‐well plate filled with double‐distilled water at room temperature. Then, the samples were taken out and rubbed with filter paper to remove the excess water. Finally, the samples were weighed (*W*_w_) at the point of swelling equilibrium. The swelling ratio was calculated according to the following formula:
(1) $$\mathrm{SR}\left(\%\right)=\frac{\left(W_{w}-W_{d}\right)}{W_{d}}\times 100\%$$

Where *W*_d_ represents the weights of freeze‐dried hydrogel samples, *W*_w_ stands for the weights of hydrogels after absorbing water.

4. *In Vitro Degradation*: The degradable properties of SA/Gel hydrogel with different concentrations of rGO were examined via quantifying the mass remaining in vitro. These samples were immersed in double‐distilled water and lyophilized for 7, 14, and 21 days. Then, the lyophilized samples were weighed. W_I_ was the initial dry weights of the sample and W_T_ was the weights after T days of immersion in water (T = 7, 14, and 21 days). Finally, the remaining weights were calculated by the following formula. These experiments were performed in triplicates and repeated thrice.
(2) $$\mathrm{Mass}\;\mathrm{remaining}\;\%=W_{T}/W_{I}\times 100\%$$

### Scanning Electron Microscope (SEM)

The morphology of the hybrid hydrogel samples was observed by SEM. Briefly, the samples were prepared in a 24‐well plate (0.4 cm in height), frozen at 4 °C and −80 °C for 2 h, respectively, and then lyophilized overnight in a vacuum freeze‐dryer (SCIENTZ‐10N, Ningbo, China). Next, the freeze‐dried hydrogel samples were cross‐linked with 5% CaCl_2_ for 30 min and washed thoroughly with sterile water. Then, they were freeze‐dried and stored in a vacuum container. The distribution of pore size and porosity of hydrogels was counted using Image J visualization software (National Institutes of Health, Bethesda, MD, USA) (*n* = 10) from SEM images.

### Cell Culture

Human fibroblasts were obtained from the human foreskin dermal tissue from the National Collection of Authenticated Cell Culture. The cells were cultured in a T‐75 flask (Corning, USA) with high‐glucose Dulbecco's modified Eagle's medium (DMEM, Hyclone, UT), and supplemented with 10% fetal bovine serum (FBS, Gibco, USA) and 1% penicillin‐streptomycin in an incubator (37°C, 5% CO_2_). The complete medium was updated every 2 days. After 85–90% confluence, the passage of 10 to 15 cells was used for subsequent experiments. The growth and proliferation of cells were observed with an inverted phase contrast microscope (CKX53, Olympus, Japan).

### In Vitro Cell Biocompatibility Measurements

Fibroblasts were used to test cell adhesion and proliferation on the hydrogel. The cells at a density of 5 × 10^4^ cells mL^−1^ were seeded on the surface of SA/Gel hydrogel. Cell viability was identified by 2 µm Calcein AM (live cell stain, 490 nm) (Life Technologies, USA) and 4 µm propidium iodides (dead cell stain, 535 nm) (PI, Life Technologies, USA). The living and dead cells were marked in green and red, respectively. Samples were washed three times by PBS and stained with Calcein AM and PI for 30 min in the dark. Then, they were washed with PBS solution. A fluorescence microscope with an imaging system (IX73, Olympus, Japan) was used for image acquisition. The cell cytotoxicity of rGO for fibroblasts was evaluated by the CCK‐8 (Dojindo, Japan) at days 1, 4, and 7 after seeding according to the protocol. Briefly, fibroblasts seeded on hydrogel with or without rGO were washed with PBS three times. Then, 100 µL of CCK‐8 and 1 mL of complete culture medium were added to each well, followed by incubation in the dark for 4 h at 37 °C. 200 µL of the medium was transferred into a 96‐well plate after incubation. Each group contained five replicate wells. The CCK‐8 medium was used as the black control. The optical density (OD) was read by the multifunctional enzyme marker (Varioskan Flash, Thermo Fisher Scientific, USA) at 450 nm. five samples were tested for each group.

Cell Migration: Wound scratch assay was used for the cell migration study. The fibroblasts at the density were 2 × 10^5^ cells well^−1^ were seeded on the 24‐well plate. A scratch wound was formed using a pipette tip of 1000 µL when the confluence of cells reached 80–90%. Then, cells were washed with PBS. The distance of scratches was observed and recorded with an inverted phase contrast microscope (*W*_d0_). The rGO dispersion in a certain volume ratio blended with the complete medium was added to the wells. The cells were cultured for 24 h and observed under the microscope again. And the distance of scratches (*W*_dt_) was recorded. Wound contraction was quantified from the images by the following equation. Experiments were repeated in triplicates.

(3) $$\mathrm{Wound}\;\mathrm{contraction}\left(\%\right)=\frac{\left(W_{d0}-W_{\mathrm{dt}}\right)}{W_{d0}}\times 100\%$$

### 4.1 Printing of Acellular SA/Gel/rGO Patches

In this study, the SA/Gel/rGO patches were printed using the Organ Printing United system (OPUS, Novaprint Therapeutics Suzhou Co., Ltd, Suzhou, China); it includes multi‐nozzle printing and multi‐material mixing. The patch model was a lattice‐rod structure with 15 mm ×15 mm × 0.75 mm, which was converted into a G‐Code file using Slic3r with the layer height of 0.15 mm (5 layers), the pore size of 1.5 mm. An inner diameter of 200 µm of the printing nozzle was selected. The ink solution was liquid at 37 °C and introduced into a special printing syringe (3 cc), but it cannot be printed in this state. Therefore, the printing syringe with the ink was placed and cooled at 4 °C for 8–10 min. And the temperature of the printer chamber was set to 19 °C. The SA/Gel/rGO patches were printed in a layer‐by‐layer deposition fashion on the printer platform. After printing each patch, they were cooled to −80 °C. Briefly, the above printed acellular patches (15 mm × 15 mm) were frozen at −80 °C, and then lyophilized overnight in a vacuum freeze‐dryer. Next, the freeze‐dried patches were cross‐linked with 5% CaCl_2_ for 30 min and washed thoroughly with sterile water. The patches were freeze‐dried again and stored in a vacuum container.

### Patch Mechanics

Multifunctional materials testing machine (HD‐5000B, Yangzhou, China) was used to characterize the tensile behavior of the SA/Gel/rGO patches. The SA/Gel patch was used as the control material. The patch samples of longitudinal strips with a length of 50 mm and a width of 20 mm were prepared. Then, the distance between the clamps was measured. The patches were stretched at a speed of 10 mm per minute at room temperature until reaching their breaking points. Five samples were measured in each group, and the average value and standard deviation were calculated. The final tensile strength was calculated in the following formula.

(4) δ=F/S

where *δ* was the tensile strength, *F* was the maximum force at the breaking points and *S* was the cross‐sectional area of patch.

### Cell Morphologies on Patches

The patches after the final freeze‐drying were sterilized with UV for 8 h and put on a 6‐well plate. Fibroblasts were seeded onto the patches at a density of 2 × 10^5^ cells cm^−2^ and incubated at 5% CO_2_ and 37 °C for 3 days. Additionally, the cell culture medium was updated every 2 days. Then, the cells with patches were fixed in 2.5% glutaraldehyde for 2 h at room temperature and then rinsed with PBS. The patches were then vacuum‐dried. The patches were sputter‐coated with gold for 30 sec and examined under a scanning electron microscope at 15 kV (SU8100, Hitachi, Japan).

### Protein Detection

The total protein of fibroblasts was extracted using ice‐cold Cell & Tissue Protein Extraction Reagent (Kang Chen. Shanghai, China). Then, according to the manufacturer's protocol, the protein concentration was determined using BCA Protein Assay Kit (Kang Chen, Shanghai, China). Briefly, protein array membranes were blocked in blocking buffer for 30 min and incubated with samples at room temperature for 2 h. Then samples were decanted, and membranes were washed. Next, membranes were incubated with diluted biotin‐conjugated antibodies at room temperature for 2 h. The membranes were washed with washing buffer and reacted with HRP‐conjugated streptavidin (1:1000 dilution) at room temperature for 2 h. Membranes were washed thoroughly and exposed to detection buffer in the dark. After acting to detect buffer, the membranes were exposed to X‐ray film. The image was developed using a film scanner. Relative expression levels of cytokines were made by comparing the signal intensities and quantified by densitometry. Positive controls were used to standardize the results from different membranes. And fold changes were calculated in protein expression.

### Gene Ontology and KEGG Enrichment Analysis

Gene ontology (http://www.geneontology.org) a systematical approach for gene and protein annotation in terms of biological process, molecular process, aisnd cellular component.^[^ ^54^ ^]^ Kyoto Encyclopedia of Genes and Genomes (KEGG; http://www.genome.jp/kegg/) is an online database depositing biological pathways of genes and biochemicals. The enriched GO terms and KEGG pathways were annotated using the R package cluster Profiler.

### Urethroplasty and Postoperative Examinations in Rabbit

To verify the biocompatibility of the 3D printed SA/Gel/rGO patches in vivo, the patches were placed in the urethral defect of rabbits for in vivo experiments. Nine adult New Zealand white rabbits had an average weight of ≈2.5 kg and were randomly divided into three groups for the urethral defect and subsequent urethroplasty. Rabbits in group 1 (*n* = 3) were repaired with the SA/Gel patch. Rabbits in group 2 (*n* = 3) were repaired with the SA/Gel/rGO_0.05_ patch. Rabbits in group 3 (*n* = 3) were repaired with the SA/Gel/rGO_0.2_ patch. These rabbits were first performed by general anesthesia with intravenous injection of pentobarbital, then the rabbit's urethras were disinfected with alcohol. The skins were sectioned at ≈3 cm proximal to the external urethral orifices, and the urethral lumens were exposed. Ventral urethral defects with a mean length × width of 2.0 cm × 0.8 cm were created in the anterior urethra of rabbits. The scaffolds with a length × width of 1.5 cm × 0.5 cm were sutured to the edge of the defect using 6‐0 absorbable polyglactin sutures (Ethicon, USA), and the whole urethral defects were sutured with 6‐0 absorbable polyglactin sutures. The rabbits were observed twice a day.

To observe the urethral leakage and stricture of the three groups of rabbits, the contrast solution was injected into the urethral lumens at 4‐ and 8‐weeks post‐surgery. Meanwhile, the rabbits were undertaken contrast‐enhanced urethrogram tests to check the condition of lumen in the urethra at 4‐ and 8‐weeks post‐surgery. The rabbits were euthanized after retrograde urethrograms at 4‐ and 8‐ weeks, and the urethral tissue for the following histology staining was collected. All the animal experiments were in accordance with the guidelines for animal care. The animal protocol (SYXK 2017‐0240) was approved by the Institutional Animal Care and Use Committee of the Shanghai Jiao Tong University Affiliated Sixth People's Hospital.

### Histology Assessment and Immunofluorescence

The urethras were harvested after 4 weeks and 8 weeks postoperatively for histology analysis. Hematoxylin and eosin staining (H&E) and Masson's trichrome staining tests were conducted to identify the epithelial layer and collagen distribution of urethra. The specimens were fixed in 4% paraformaldehyde for 30 min at room temperature. Then, they were dehydrated with different grades of alcohol and embedded in the paraffin blocks. Histological sections were prepared and observed using an optical microscope. To further demonstrate the repair of urethral function, the samples were stained for immunofluorescence for epithelial cytokeratin AE1/AE3 (Santa Cruz Biotechnology, Inc.), CD31 (blood vessels, 1:100, Proteintech Group, Inc), CD206 (1:500, Proteintech Group, Inc), and PCNA (Proliferating Cell Nuclear Antigen 1:500, Abcam plc). Nuclei were stained with DAPI (1:500, Life Technologies). Afterward, the specimens were imaged and observed by an optical microscope.

### Flow cytometry

After 85–90% confluence, leukemia cells in mouse macrophage (RAW 264.7 cells) were digested with pancreatic enzyme, and centrifuged at 1000 rpm for 5 min. Then the supernatant was removed, and cells were resuspended with a complete medium. RAW264.7 cells were seeded in 6‐well plates (5 × 10^5^ cells/well), and placed in a CO_2_ incubator at 37 °C overnight. After 1 day, cells were divided into two groups when the cell density reached ≈40–50%. Group 1 (Control; 0.02rGO; 0.1rGO) was temporarily not treated. Group 2 (Control; M1; M1 + 0.02rGO; M1 + 0.1rGO), where M1; M1 + 0.02rGO; M1+ 0.1rGO, first treated with LPS (100 ng/mL) + INF‐r (50 ng mL^−1^) for 24 h to induce M1 type. On the third day, when the cell density reaches ≈70–80%, the corresponding concentration of rGO was added to the two groups, which were 0.02rGO, 0.1rGO, and treated for 24 h. RAW264.7 cells in the two groups of plates were collected separately for flow cytometry and RT‐PCR detection after 24 h. Flow cytometry was used to determine the effect of graphene on the polarization of Raw264.7 to M1, M2. Fluorescently labeled antibodies CD68, CD86, and CD206 were added to both groups of cells. Before staining, Raw264.7 cells were fixed and broken, then mixed with antibodies and incubated at 4 °C for 30 min in the dark. Then, the cells were resuspended with a cell staining buffer, followed by centrifugation for 5 min at 300 g, and discard the supernatant. Finally, the cells were resuspended by 200 µL cell stained buffer for detection and analysis with flow cytometry (Sysmex, CyFlow Cube8).

### RT‐PCR

Relative quantification for mRNA gene expression studies. Extraction of mRNA with Invitrogen's trizol. The extracted mRNA was then reverse‐transcribed to become a cDNA template. Finally, a real‐time PCR experiment was performed using cDNA as a template.

### Statistical Analysis

All the statistical significance for the experiments was analyzed using the one‐way ANOVA and paired‐samples t‐test. The error bar represents the mean ± standard deviation (SD) of measurements performed on each sample group. It was a statistically significant difference based on *p* <0.05 (*p <0.05, **p <0.01, ***p <0.001, and ****p <0.0001).

## Conflict of Interest

The authors declare no conflict of interest.

## Supporting information

Supporting Information

## Data Availability

Research data are not shared.
