# Supplementary material for: Urethral Microenvironment Adapted Sodium Alginate/Gelatin/Reduced Graphene Oxide Biomimetic Patch Improves Scarless Urethral Regeneration
Source: Adv Sci (Weinh). 2023 Nov 16;11(2):2302574. doi: 10.1002/advs.202302574 (PMC10787096; doi:10.1002/advs.202302574)
Supplement: Supplementary file 1 — Supporting Information [file ADVS-11-2302574-s001.pdf]

ADVANCED SCIENCE
Open Access

Supporting Information

for *Adv. Sci.*, DOI 10.1002/advs.202302574

Urethral Microenvironment Adapted Sodium Alginate/Gelatin/Reduced Graphene Oxide Biomimetic Patch Improves Scarless Urethral Regeneration

Liyang Wang, Kai Wang, Ming Yang, Xi Yang, Danyang Li, Meng Liu, Changmei Niu, Weixin Zhao, Wenyao Li, Qiang Fu* and Kaile Zhang**

Urethral Microenvironment Adapted Sodium Alginate/Gelatin/Reduced Graphene Oxide Biomimetic Patch Improves Scarless Urethral Regeneration

Liyang Wang, [a, b ,1] *Kai Wang,* [f ,1] *Ming Yang,* [a, d ,1] *Xi Yang,* [c,] *Danyang Li,* [a, b] *Meng Liu,* [a, d] *Changmei Niu,* [c] *Weixin Zhao,* [e] *Wenyao Li,* *[b] *Qiang Fu,* *[a, d] *Kaile Zhang,* *[a, d]

L. Y. Wang, M. Yang, D. Y. Li, M. Liu, Dr. Q. Fu, Dr. K. L. Zhang

[a] The Department of Urology, Shanghai Sixth People's Hospital Affiliated to Shanghai Jiao Tong University School of Medicine, Shanghai Jiao Tong University, Shanghai, 200233, P.R. China.

L. Y. Wang, D. Y. Li, Dr. W. Y. Li

[b] School of Materials Science and Engineering, Shanghai University of Engineering Science, Shanghai, 201620, P. R. China

X. Yang, C. M. Niu

[c] Novaprint Therapeutics Suzhou Co., Ltd, Suzhou 215000, P. R. China

M. Yang, M. Liu, Dr. Q. Fu, Dr. K. L. Zhang

[d] Shanghai Eastern Institute of Urologic Reconstruction, Shanghai 200000, P. R. China

W. X. Zhao

[e] Wake Forest Institute for Regenerative Medicine, Winston-Salem, NC, 27155, United States

K. Wang

[f] Clinical Research Center, Shanghai Chest Hospital, Shanghai Jiao Tong University, Shanghai, 200233, P. R. China

*Corresponding Authors: Kaile Zhang (great_z0313@126.com), Qiang Fu (jamesqfu@126.com), Wenyao Li (liwenyao@sues.edu.cn)

[1] These authors contributed equally to this work.

Table S1. Chemicals used for Gr composite hydrogel preparation.

Groups	SA (mg)	Gel (mg)	rGO (mL)	sterile water (mL)
SA/Gel	400	400	0	20.0
SA/Gel/$rGO_{0.02}$	400	400	0.4	19.6
SA/Gel/$rGO_{0.05}$	400	400	1.0	19.0
SA/Gel/$rGO_{0.1}$	400	400	2.0	18.0
SA/Gel/$rGO_{0.2}$	400	400	4.0	16.0

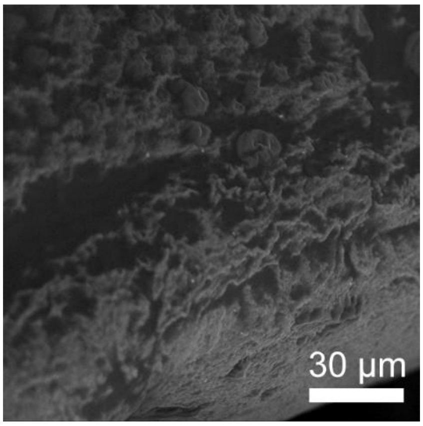

Figure S1 SEM image of the urethral decellularized matrix.

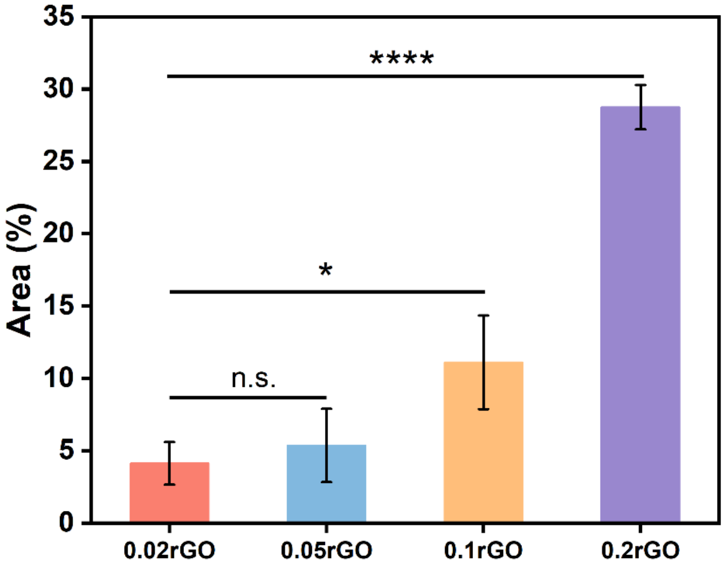

Figure S2 Area rates (%) of rGO at various concentrations (*p <0.05, ****p <0.0001, n.s. means not significant).

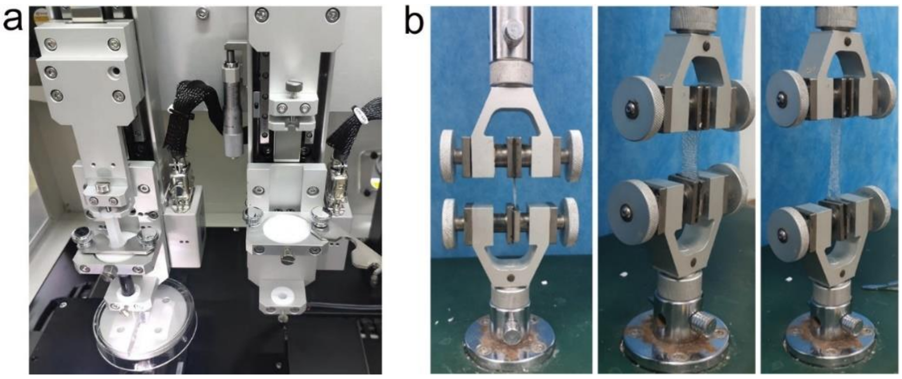

Figure S3 (a) The 3D printer for fabricating the patches. (b) Process of stretching the patch by the mechanical testing machine.

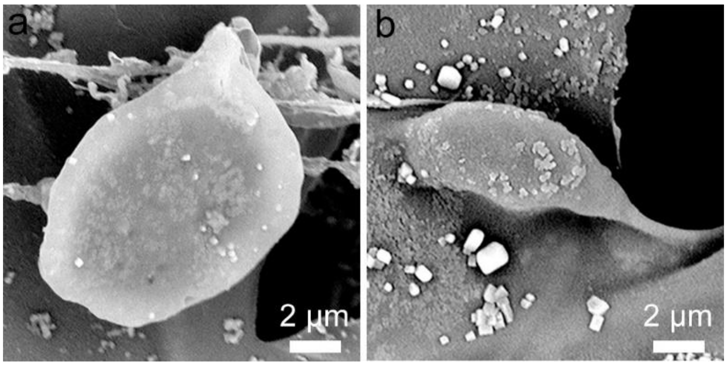

Figure S4 SEM of the morphology of fibroblasts on SA/Gel/rGO patches. (a-b) The different morphology of fibroblasts.

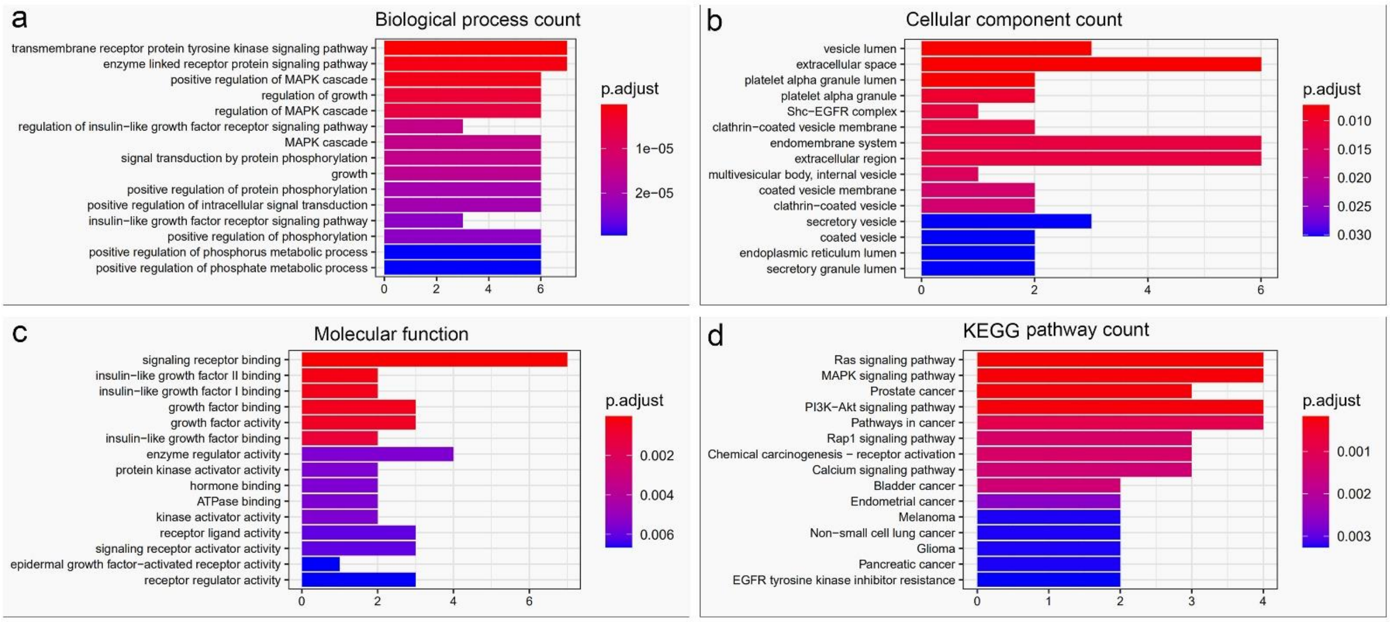

Figure S5 GO-level sequence distribution in bar plot for (a) biological process, (b) cellular component, (c) molecular process, and (d) KEGG pathways of 0 versus $rGO_{0.02}$ group. The longer bars indicate the more enriched genes. The deeper red color suggests the more obvious difference.

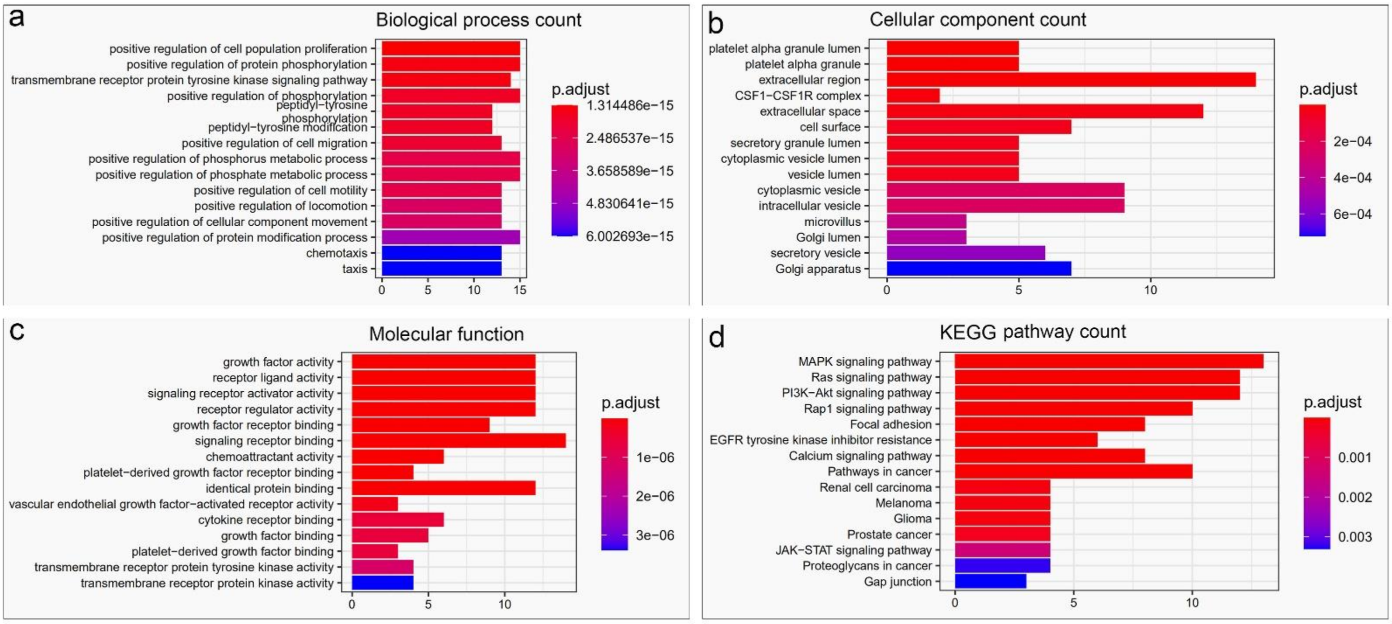

Figure S6 GO-level sequence distribution in bar plot for (a) biological process, (b) cellular component, (c) molecular process, and (d) KEGG pathways of 0 versus $rGO_{0.05}$ group. The longer bars indicate the more enriched genes. The deeper red color suggests the more obvious difference.

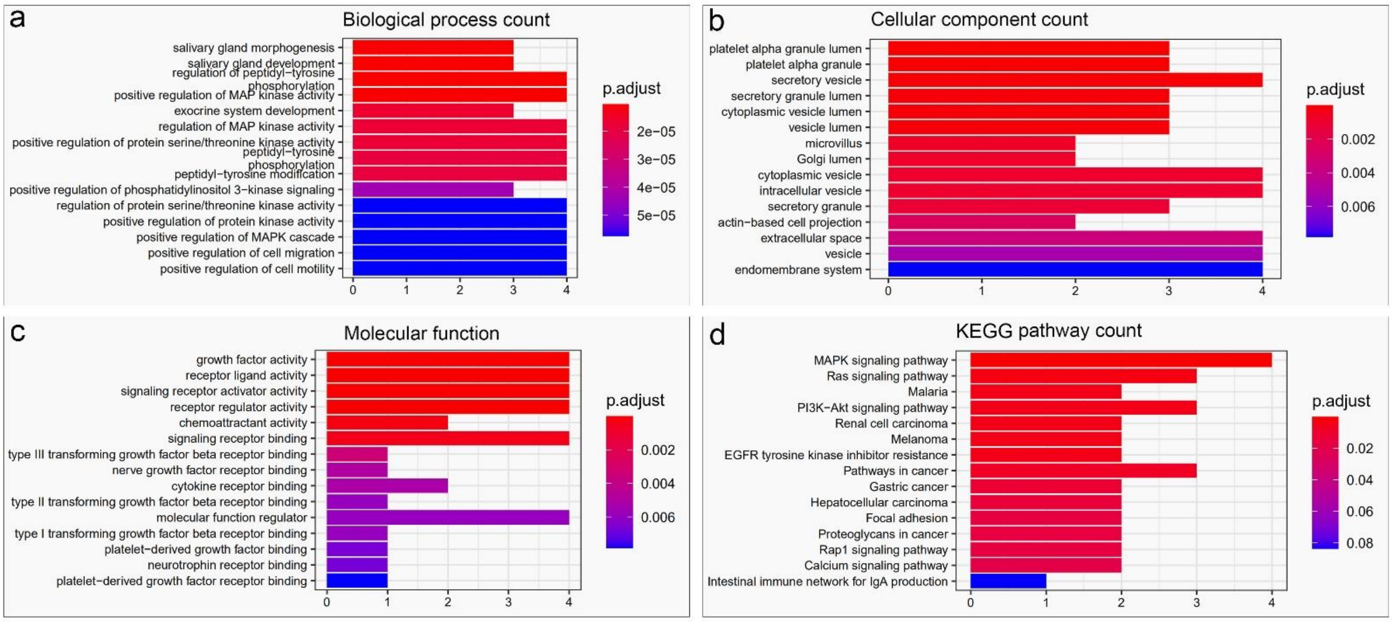

Figure S7 GO-level sequence distribution in bar plot for (a) biological process, (b) cellular component, (c) molecular process, and (d) KEGG pathways of 0 versus $rGO_{0.1}$ group. The longer bars indicate the more enriched genes. The deeper red color suggests the more obvious difference.

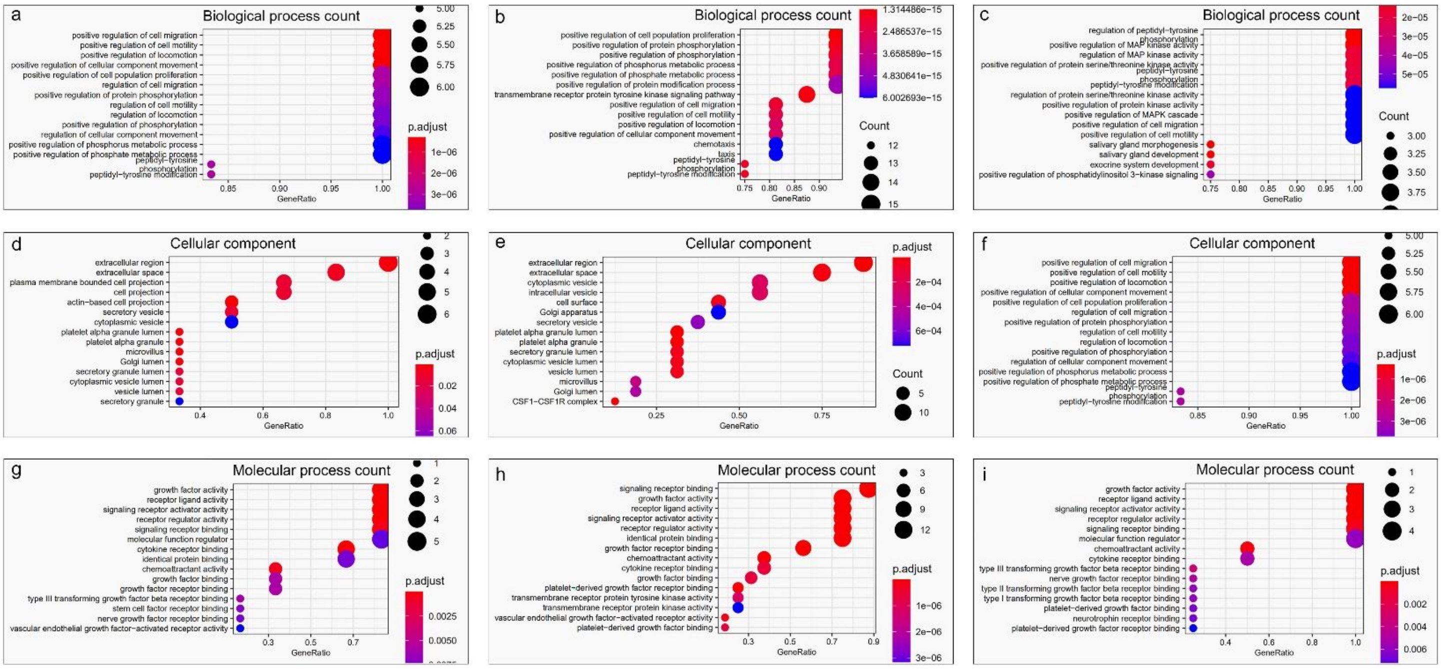

Figure S8 Functional analysis based on the DEGs. Bubble graph for GO enrichment for Biological process, Molecular process and Cellular component of 0 *v.s.*$rGO_{0.02}$ group (a, d, g), 0 *v.s.*$rGO_{0.05}$ group (b, e, h) and 0 *v.s.*$rGO_{0.1}$ group (c, f, i). The bigger bubble means more genes are enriched, and the increasing depth of red means the differences were more obvious; q-value: the adjusted p-value.

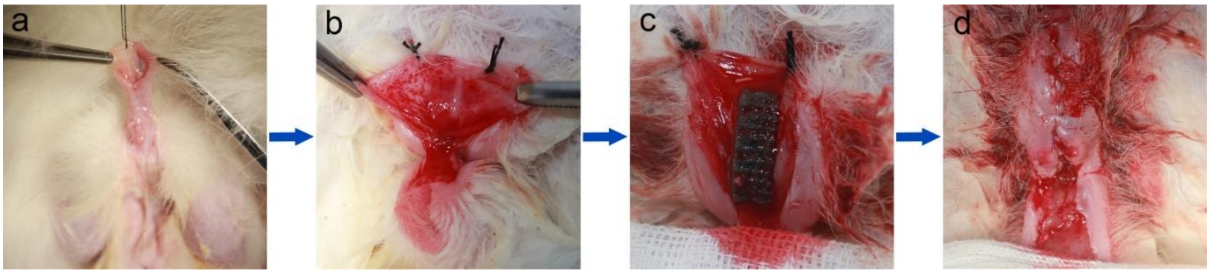

Figure S9 Surgical procedures for urethral trauma modeling and repair. (a) The skin was sectioned at approximately 3 cm proximal to the external urethral orifice. (b) The urethra lumen

was exposed, and the mucosal layer was destroyed. (c) The patches were sutured to the dorsal urethral wound. (d) The whole urethra defects were sutured.
